# Supplementary material for: Panicle Apical Abortion 7 Regulates Panicle Development in Rice (Oryza sativa L.)
Source: Int J Mol Sci. 2022 Aug 22;23(16):9487. doi: 10.3390/ijms23169487 (PMC9409353; doi:10.3390/ijms23169487)
Supplement: Supplementary file 1 [file ijms-23-09487-s001.zip › ijms-1808710-supplementary.pdf]

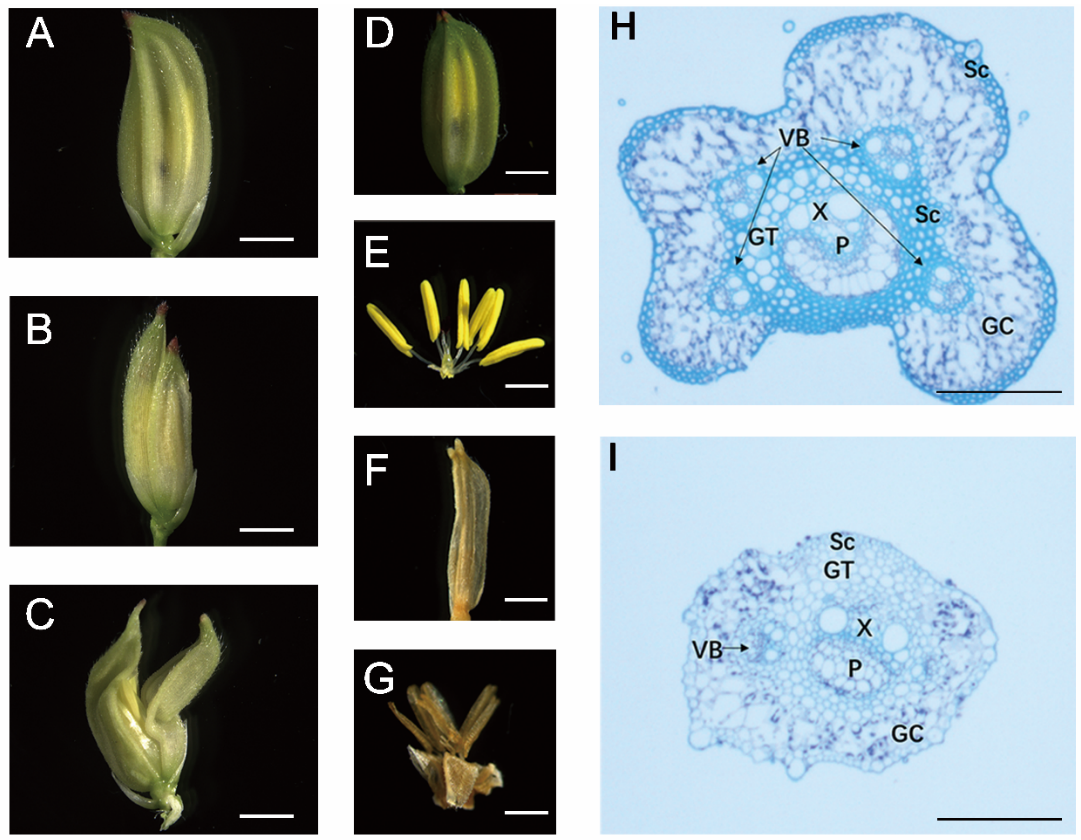

Figure S1 Observations of spikelets and pedicels in WT and *paa7*.

(A-G) Spikelets or floral organs in *paa7* (A-C, F, G) and WT (D, E), Bars =2mm. (B-C) Transverse sections of pedicels of WT (B) and *paa7* mutant. (C) of 12cm panicle, GC: green cells; GT: ground tissue; P: phloem; Sc: sclerenchyma VB: vascular bundle; X: xylem; Bars=100μm.

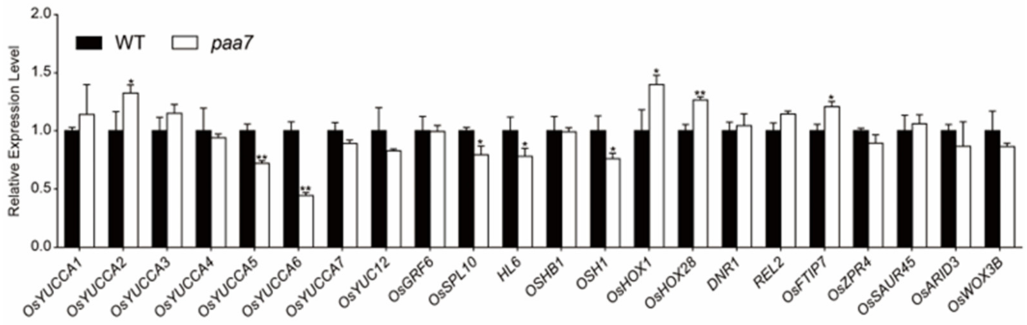

Figure S2 Expression level of genes associated with auxin synthesis

Expression level of genes involved the auxin synthesis pathway in 7cm panicles of WT and *paa7,* Data are shown as means ± SD from 3 replicates. Double and single Asterisks indicate statistically significant differences compared to the WT at $p < 0.01$ and $p < 0.05$, respectively, Student's *t*-test.

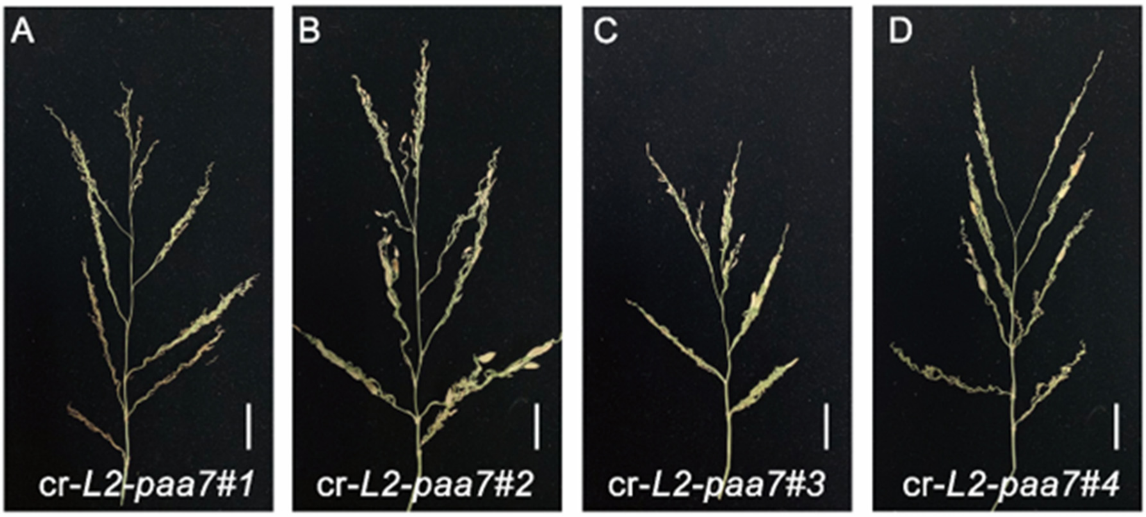

Figure S3 Unfolded phenotype of transgenic plants.
Panicle of *cr-L2-paa7#1* (A), cr-L2-paa7#2 (B), cr-L2-paa7#3 (C) and cr-L2-paa7#4(D), Bars =2 cm.

Table S1. Genetic analysis of the *paa7* phenotype

Cross	Year	F_1	No. of F_2 Individuals		χ^2
			Wild type	Mutant Type	
paa7×II-32B	2017	Wild type	460	159	0.1529
paa7×9311		Wild type	989	349	0.815
paa7×II-32B	2018	Wild type	4003	1405	2.701
paa7×9311		Wild type	2788	872	2.7836

Table S2. Phenotype statistics of derivation of *paa7* and *lax2-4* mutant

Cross	F_1	No. of F_2 Individuals				χ^2(9:3:3:1)
		Wild Type	*paa7* Type	*lax2-4* Type	*ap1* Type	

paa7×lax2-4	Wild type	287	97	95	37	0.77
lax2-4×paa7	Wild type	259	82	88	24	0.94

Table S3 List of primer names and corresponding sequences used in this study

Primer name	Primer sequence (5'-3')	Experiments
D20-F	GATCGCCTTGGATTAAGAACAAGA	Fine mapping
D20-R	AATTTGAAGCCGTGGAGGAGA	Fine mapping
I12-F	ACCTTGCACTTTACCTTGCCT	Fine mapping
I12-R	TGTAGGATCAAGGAGGCCGA	Fine mapping
D25-F	CCCACCGTTCGTTGGTTTG	Fine mapping
D25-R	ATGGAGGAGGACTAGGGCTTT	Fine mapping
D31-F	TTACCCGGCACCGATTTCTC	Fine mapping
D31-R	ACACTGGAAGCTACACTGGAAG	Fine mapping
com-fzp-f	gctcggtacccggggatccCCGATGTCCCCTGAAAATCA	Vectors construction
com-fzp-r	aggtcgactctagaggatccATCCACCACCTGCGTGTTAT	Vectors construction
OE-fzp-f	ttctgcaggagctcggtaccATGAACACTCGAGGCAG	Vectors construction
OE-fzp-r	tcgctcatggatccggtaccTGAATGGAGAGTAGGAGTC	Vectors construction
FZP-OsU3T1F	ggcaTATATGCATGATCGCTCAG	Vectors construction
FZP-OsU3T1R	aaacCTGAGCGATCATGCATATA	Vectors construction
lax2U3T2F	ggcaCATTCTTGCCTCAGATTCCA	Vectors construction
lax2U3T2R	aaacTGGAATCTGAGGCAAGAATG	Vectors construction
U-F	CTCCGTTTTACCTGTGGAATCG	Vectors construction
gRNA-R	CGGAGGAAAATTCCATCCAC	Vectors construction
Uctcg-B1'	TTCAGAggtctcTctcgCACTGGAATCGGCAGCAAAGG	Vectors construction
gRcggt-BL	AGCGTGggtctcGaccgGGTCCATCCACTCCAAGCTC	Vectors construction
Ubiquitin-realF	aaccagctgaggcccaaga	qRT-PCR
Ubiquitin-realR	acgattgatttaaccagtccatga	qRT-PCR
FZP-realF	cagggctccgactcctactct	qRT-PCR
FZP-realR	caatgcccggtgcaactc	qRT-PCR
OsYUCCA1-realF	CGCAACACGGTGCATGTC	qRT-PCR
OsYUCCA1-realR	CGATGCCGAACGTGGATAG	qRT-PCR
OsYUCCA2-realF	TTGCAATGTGGCTGCTCAA	qRT-PCR
OsYUCCA2-realR	AAGCAGTAAAATCCGGTCTACGA	qRT-PCR
OsYUCCA3-realF	TCTTGACGTCGGCACATTTG	qRT-PCR
OsYUCCA3-realR	ATGGCCGGCCGTACCT	qRT-PCR
OsYUCCA4-realF	AAGGTGACCCCAGCAATACAA	qRT-PCR
OsYUCCA4-realR	AGTGCTGCCATCCACAAACTC	qRT-PCR
OsYUCCA5-realF	CGTCAGGGACAAGATTCATGTTC	qRT-PCR
OsYUCCA5-realR	CTGAGAGGCCAAATGTTGAGATG	qRT-PCR
OsYUCCA6-realF	ACCGGATACCAAAGCAACGT	qRT-PCR
OsYUCCA6-realR	CCCCTCTTGTGTGAAGAAGTCACT	qRT-PCR
OsYUCCA7-realF	AGCAATGTGCCCCAATGG	qRT-PCR
OsYUCCA7-realR	TGGGTACCCGTCCTTGTTGA	qRT-PCR

OsYUC12F	CATAGTTGTTCGCAGCCC	qRT-PCR
OsYUC12R	CATTACAAGAAGAGGGTCC	qRT-PCR
OsGRF6F	CTTGCTCTCTGCAATATAGC	qRT-PCR
OsGRF6R	GAGAACGGAAACAGGCTG	qRT-PCR
OsSPL10F	CTGCCAGCAATGCAGCAG	qRT-PCR
OsSPL10R	CGTCGCCGCCGCCGCCGG	qRT-PCR
HL6F	CACATCTGCACCTCCACTTG	qRT-PCR
HL6R	GATCCTCCGGGGCAGCTGCC	qRT-PCR
OSHB1F	TGGTTCCAGAACAGAAG	qRT-PCR
OSHB1R	GAGACCTGCTTCTGGAGTC	qRT-PCR
OSH1F	CATGGCGCACACGCCGC	qRT-PCR
OSH1R	CGCCGAGGACGCCGAGC	qRT-PCR
OsHOX1F	CTTCTCCTCGTCTTCCTC	qRT-PCR
OsHOX1R	CTGCCGCCGGCGCACGGTTC	qRT-PCR
OsHOX28F	GACGCACCACACCCTCAC	qRT-PCR
OsHOX28R	CACCTCCGTCTGCTTCAGC	qRT-PCR
DNR1F	GAAGCACTTCTCGAGAAG	qRT-PCR
DNR1R	GGTGAGGACCACATTTAC	qRT-PCR
REL2F	CAGGAGGTCAAGGTAATTC	qRT-PCR
REL2R	CTTGGTCTTATCCAGCTTG	qRT-PCR
OsFTIP7F	CAGCCAGTGGTACAATCTTG	qRT-PCR
OsFTIP7R	GCTGTAATGTGTTGACTC	qRT-PCR
OsZPR4F	GAGAGGGTGAACCTGAAGC	qRT-PCR
OsZPR4R	GTTGGTGTTGAGCTTGGCG	qRT-PCR
OsSAUR45F	CAGTGCCAGGAGACTTG	qRT-PCR
OsSAUR45R	CTGCTACAGATGTGCAGC	qRT-PCR
OsARID3F	GAAGGGTTGAATTGCCTC	qRT-PCR
OsARID3R	CTTCTCATAGAAGTTACGG	qRT-PCR
OsWOX3BF	CACCGCCGCCGCCGCAC	qRT-PCR
OsWOX3BR	GACGAACGCCCGATCGCC	qRT-PCR
(PL)YUCCA2-F	atctgtcgacctcgagAGGAAAACCAGGAG	Yeast one hybrid
(PL)YUCCA2-R	gagcacatgcctcgagCTTTGATATGCTTG	Yeast one hybrid
(PL)YUCCA5-R	gagcacatgcctcgagCCATCTTGTTGCGAGGTT	Yeast one hybrid
(PL)YUCCA6-F	atctgtcgacctcgagTGCTCCACTCCACTGATG	Yeast one hybrid
(PL)YUCCA6-R	gagcacatgcctcgagGAGGTGACGGACTGGTTG	Yeast one hybrid
(PL)OsSPL10F	atctgtcgacctcgagGGCATGCACAAATCCC	Yeast one hybrid
(PL)OsSPL10R	gagcacatgcctcgagGCCCGCCTCCTCCTCG	Yeast one hybrid
(PL)HL6F	atctgtcgacctcgagCTAAAGTGCAACCATG	Yeast one hybrid
(PL)HL6R	gagcacatgcctcgagGGCGATAGATATGATC	Yeast one hybrid
(PL)OSH1F	atctgtcgacctcgagGAGGTACAGAGGTTC	Yeast one hybrid
(PL)OSH1R	gagcacatgcctcgagGAGAGAAGCTCAAGAC	Yeast one hybrid
(PL)OsHOX1F	atctgtcgacctcgagGTGGTCAAAAGTGAG	Yeast one hybrid
(PL)OsHOX1R	gagcacatgcctcgagTGATTCCTCTCAACTC	Yeast one hybrid
(PL)OsHOX28F	atctgtcgacctcgagCACCCGGGACCAAC	Yeast one hybrid

(PL)OsHOX28R	gagcacatgcctcgagGTCTTAAGTGCTCTAG	Yeast one hybrid
(PL)OsFTIP7F	atctgtcgacctcgagCGGTAGAGATTCGG	Yeast one hybrid
(PL)OsFTIP7R	gagcacatgcctcgagGAACGAAATTCTTG	Yeast one hybrid
chip-YUC6-F1	CACTAATTTTATTTGGCTAC	ChIP-qPCR
chip-YUC6-R1	TGATGTTTTGGTGTTGAA	ChIP-qPCR
chip-YUC6-F2	CCACAATTCCACTTCTAT	ChIP-qPCR
chip-YUC6-R2	TGGCACCATTGTCTCACT	ChIP-qPCR
chip-YUC6-R3	AGACAATGGTGCCAATCA	ChIP-qPCR
chip-YUC6-F3	CAGCCAATAATGCTAAAACA	ChIP-qPCR
chip-YUC6-F4	TTAGCATTATTGGCTGTA	ChIP-qPCR
chip-YUC6-R4	GTTCTGGCATTGGCTCTA	ChIP-qPCR
chip-YUC6-F5	TAGTACGGTAGAGCCAATG	ChIP-qPCR
chip-YUC6-R5	AACTTTTATGCACGAATC	ChIP-qPCR
chip-YUC6-R6	GGAAGCACTTATACCTCT	ChIP-qPCR
chip-YUC6-F6	TACTCCCTCTGTCCCAAA	ChIP-qPCR
chip-YUC6-F7	TGGGACAGAGGGAGTAGAAA	ChIP-qPCR
chip-YUC6-R7	ACCGTTGGACATCGGCAC	ChIP-qPCR
chip-YUC6-F8	GATGTCCAACGGTCCAAG	ChIP-qPCR
chip-YUC6-R8	GTGTAGCTGTGCCTGTGC	ChIP-qPCR
chip-YUC6-F9	GCACAGGCACAGCTACAC	ChIP-qPCR
chip-YUC6-R9	GCAAGGCAAGGATGAGGA	ChIP-qPCR
chip-YUC6-F10	CCTCATCCTTGCCTTGCC	ChIP-qPCR
chip-YUC6-R10	GCCGGAGTGAGACTGTGA	ChIP-qPCR
chip-YUC6-F11	TCGCTGTCCCTCGCTCACT	ChIP-qPCR
chip-YUC6-R11	GACCACGATCGGCCCGTT	ChIP-qPCR
chip-YUC6-F3	CAGCCAATAATGCTAAAACA	ChIP-qPCR
chip-YUC6-F4	TTAGCATTATTGGCTGTA	ChIP-qPCR
chip-YUC6-R4	GTTCTGGCATTGGCTCTA	ChIP-qPCR
chip-YUC6-F5	TAGTACGGTAGAGCCAATG	ChIP-qPCR
chip-YUC6-R5	AACTTTTATGCACGAATC	ChIP-qPCR
chip-YUC6-R6	GGAAGCACTTATACCTCT	ChIP-qPCR
chip-YUC6-F6	TACTCCCTCTGTCCCAAA	ChIP-qPCR
chip-YUC6-F7	TGGGACAGAGGGAGTAGAAA	ChIP-qPCR
chip-YUC6-R7	ACCGTTGGACATCGGCAC	ChIP-qPCR
chip-YUC6-F8	GATGTCCAACGGTCCAAG	ChIP-qPCR
chip-YUC6-R8	GTGTAGCTGTGCCTGTGC	ChIP-qPCR
chip-YUC6-F9	GCACAGGCACAGCTACAC	ChIP-qPCR
chip-YUC6-R9	GCAAGGCAAGGATGAGGA	ChIP-qPCR
chip-YUC6-F10	CCTCATCCTTGCCTTGCC	ChIP-qPCR
chip-YUC6-R10	GCCGGAGTGAGACTGTGA	ChIP-qPCR
chip-YUC6-F11	TCGCTGTCCCTCGCTCACT	ChIP-qPCR
chip-YUC6-R11	GACCACGATCGGCCCGTT	ChIP-qPCR
YUCCA6-ES-F	GTAGGGTCGAAAGACGGTGCCGATGTCCAACGGTCCA	EMSA
	AGACATAGAGTGAAAAAGAAAG	EMSA

YUCCA6-ES-R	CTTTCTTTTTCACTCTATGTCTTGGACCGTTGGACATCG	EMSA
	GCACCGTCTTTCGACCCTAC	EMSA
(BK)LAX2/199-F	atggaggccgaattcATGCCGCCGCCGGGATCC	Yeast two hybrid
(BK)LAX2/338-R	gatccccgggaattcCTACTCTGATTCATCTTC	Yeast two hybrid
(AD)LAX2/199-F	gaggccagtgaattcATGAACACTCGAGGCAG	Yeast two hybrid
(AD)LAX2/338-R	acccgggtggaattcTCAATGGGAGAGGAAGC	Yeast two hybrid
(YC)LAX2-F	GGAGCTCGGTACCCGGGATGGTCCCGGCTCGG	BiFC
(YC)LAX2-R	GTATGGGTACATACTAGTGGGCCTTCTGCCATAC	BiFC
(YN)FZP-F	GAGGAGGATCTTCCCGGGAACACTCGAGGCAGCGGC	BiFC
(YN)FZP-R	ATGCCTGCAGGTCGACTCAATGGGAGAGGAAGC	BiFC
MBP-PAA7-F	ttcggatcctctagagtcgacATGAACACTCGAGGCAGC	Pull-down
MBP-PAA7-R	caagcttgcctgcaggtcgacATGGGAGAGGAAGCTGAA	Pull-down
GST-LAX2-F	CTGGTTCCGCGTGGATCCATGCCGCCGCCGGGATCC	Pull-down
GST-LAX2-R	CGGGAATTCCGGGGATCCTGGACGGCGACCATACTG	Pull-down
GFP-PAA7-F	GGGCTGCAGGAATTCATGAACACTCGAGGCAGC	CO-IP
GFP-PAA7-R	CTTGATATCGAATTCATGGGAGAGGAAGCTGAA	CO-IP
HA-LAX2-F	GCTCGGTACCAAGCTTATGCCGCCGCCGGGATCC	CO-IP
HA-LAX2-R	CCGCACTAGTAAGCTTGGGCCTTCTGCCATAC	CO-IP
